# Factors Affecting Hospital Stay Duration in the Conservative Treatment of Patients With Osteoporotic Vertebral Fractures

**DOI:** 10.7759/cureus.95127

**Published:** 2025-10-22

**Authors:** Koshiro Shimasaki, Kousei Miura, Tomohiro Yoshizawa, Tomofumi Nishino, Harumitsu Ichimura, Masafumi Uesugi, Hajime Mishima

**Affiliations:** 1 Department of Orthopedic Surgery, Graduate School of Comprehensive Human Sciences, University of Tsukuba, Tsukuba, JPN; 2 Department of Orthopedic Surgery, Ibaraki Seinan Medical Center Hospital, Sakai, JPN; 3 Department of Orthopedic Surgery, Faculty of Medicine, University of Tsukuba, Tsukuba, JPN

**Keywords:** cognitive function, conservative treatment, length of hospital stay, osteoporotic vertebral fracture, sarcopenia

## Abstract

Introduction: Our research question explores the factors contributing to the substantial variation in the length of hospital stay among patients receiving inpatient conservative treatment for osteoporotic vertebral fractures (OVFs). This study aimed to identify key determinants influencing hospitalization duration in this patient population.

Methods: Overall, 187 patients admitted for conservative treatment of OVF at a single institution between April 2020 and March 2024 were retrospectively analyzed. Data on age, sex, Charlson Comorbidity Index (CCI), cognitive impairment, pre-injury mobility, cohabitation status, in-hospital complications, nutritional status, sarcopenia, discharge destination, and length of stay were obtained. A subgroup analysis was conducted to compare patients discharged home with those discharged to non-home settings.

Results: Multiple regression analysis revealed that cognitive impairment, pre-injury mobility, cohabitation status, and in-hospital complications significantly influenced the length of stay. In the subgroup analysis, sex and cognitive impairment were significant in the univariate analysis, whereas cognitive function remained significant in the multivariate analysis.

Conclusions: Inpatients with OVFs undergoing conservative treatment had a significantly longer length of stay if they had cognitive impairment, lower pre-injury mobility, lived alone, or experienced hospitalization-related complications. Patients with cognitive impairment were less likely to be discharged home but more likely to be discharged to non-home settings, including care facilities. Early intervention by a multidisciplinary team targeting these factors can increase home-discharge rates, reduce length of stay, lower medical costs, and extend healthy life expectancy.

## Introduction

In Japan, the percentage of individuals ≥65 years of age is approaching 30% of the total population, leading to a super-aged society. This demographic trend is expected to continue in the foreseeable future [1]. With an aging population, an increase in the incidence of osteoporotic vertebral fractures (OVFs), which are the most common type of fragility fractures, is anticipated [2], along with a rise in the number of patients with OVFs.

Many patients with OVFs achieve favorable treatment outcomes with conservative management. However, the medical costs associated with OVFs are significantly driven by the use of surgical devices and the length of hospital stay. Compared with surgical treatment, conservative management usually results in prolonged hospital stays. The duration of hospitalization in conservative management varies widely across countries due to cultural and systemic differences in admission and discharge practices. For instance, in Italy in 2008, conservative management prolonged hospitalization by an average of 31 days. It prolonged hospitalization by 11 and 51 days in the UK in 2010 and in Japan in 2018, respectively. Moreover, the length of hospital stay in conservative management is highly complex and influenced by multiple factors, typically necessitating treatment for several complications and resulting in delayed discharge [3].

Factors contributing to the length of hospital stay in the conservative management of OVFs have not been fully elucidated. Therefore, this study aimed to investigate the factors contributing to the length of hospital stay in patients undergoing conservative treatment for OVFs. We hypothesized that prolonged hospital stays in patients undergoing conservative treatment for OVFs are influenced not only by medical reasons (such as complications during hospitalization) but also by social factors (such as cognitive impairment and living environment). The identification of factors that contribute to the length of hospital stay is crucial since it could lead to its reduction. To our knowledge, this is the first study to investigate social and patient background factors contributing to the length of hospital stay in patients undergoing conservative treatment for OVFs through a multivariate analysis.

## Materials and methods

Study design and setting

This retrospective case-control study was conducted at a single institution, Ibaraki Seinan Medical Center Hospital, Japan, which is an acute care hospital under the Diagnosis Procedure Combination (DPC) system, between April 2020 and March 2024, focusing on patients who underwent conservative inpatient management for OVFs. The study protocol was approved by the Ethics Committee of our hospital (approval number: R2024-001).

In accordance with the Ethical Guidelines for Life Science and Medical Research Involving Human Subjects, information regarding the study was publicly disclosed to provide participants or their representatives the opportunity to opt out. The opt-out document was posted on the hospital’s website (Ibaraki Seinan Medical Center Hospital Homepage: https://seinan-mch.or.jp/medicalinfo/optout.php). The data of the participants who requested to withdraw from the study were excluded from the analysis and immediately discarded.

Study samples

At our hospital, not all patients with vertebral body fractures undergo inpatient treatment. In this study, we included patients who visited the hospital’s outpatient department or were transported to the hospital due to acute low back pain following a fall from a standing position or lower-impact trauma (such as non-high-energy trauma). Among these patients, those who could not return home due to pain and required hospitalization were included. The diagnosis of fresh vertebral body fractures was confirmed using MRI, which showed high signal intensity on both T1-weighted images and short tau inversion recovery sequences within the vertebral body.

The exclusion criteria included cases that required surgical intervention during hospitalization; patients with difficulty understanding the treatment protocol and unable to adhere to bed rest, leading to early discharge; cases with missing data; and patients with severe complications or who experienced exacerbation of comorbidities, necessitating transfer to another department during hospitalization.

Exposures

Conservative management was initiated for all patients with fresh vertebral body fractures, following a protocol based on a previous study [4]. Patients were initially placed on bed rest with gradual head elevation up to 30°. Once pain with movement was alleviated, patients began wearing a rigid trunk orthosis and initiated supervised mobilization from the bed. Mobilization with a trunk orthosis was commenced within two weeks, even if the pain persisted during movement.

Data collection

Data on age (mean ± standard deviation (SD)), sex, comorbidities, cognitive impairment, pre-injury mobility, cohabitation status, hospital complications, nutritional status, skeletal muscle mass, discharge destination, and length of hospital stay (mean ± SD) were retrospectively obtained.

Comorbidities were assessed at admission using the Charlson Comorbidity Index (CCI) [5] (mean ± SD). Cognitive impairment was identified at admission using Hasegawa’s dementia scale (scores ≤20) if diagnosed with dementia and under medication [6]. Pre-injury mobility was categorized into three levels: independent walking, walking with support, and wheelchair use. Cohabitation status was assessed as living alone or living with others. Hospital complications were defined as adverse events that newly occurred after hospitalization, requiring intravenous treatment, consultation with other departments, or additional treatment from other specialties.

**Figure 1:**
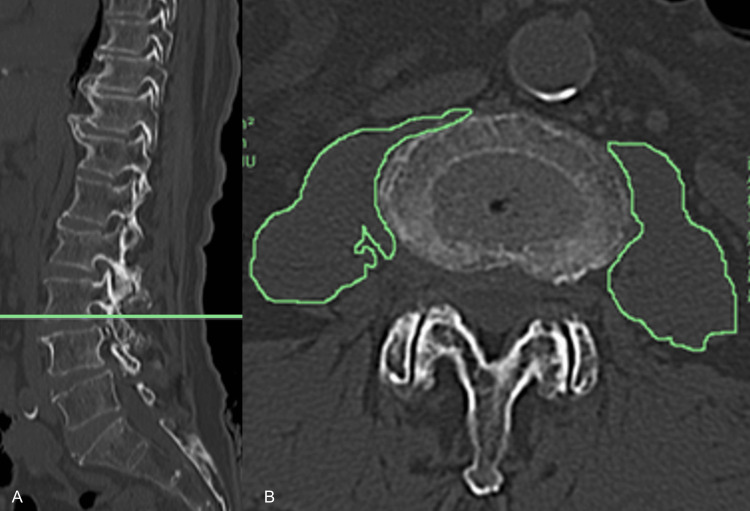
Measurement method of the cross-sectional area of the iliopsoas muscle Plain computed tomography of the thoracic and lumbar spine was performed upon admission. Panel A shows a sagittal view of the thoracolumbar spine, with a horizontal line drawn parallel to the inferior border of the third lumbar vertebral body. Panel B presents the corresponding axial image at the level indicated by the line in Panel A, in which the iliopsoas muscle was manually traced using the freehand method. All measurements were performed twice using the Picture Archiving and Communication System (Konica Minolta Medical & Graphic, Inc., Tokyo, Japan), and the average of the two values was used for analysis.

Nutritional status was assessed using the Controlling Nutritional Status (CONUT) score [7] (mean ± SD) at admission. Skeletal muscle mass was evaluated using the psoas muscle index at the level of the lower edge of the third lumbar vertebra. This was calculated by summing the bilateral psoas muscle cross-sectional areas and dividing the sum by height squared. Sarcopenia was determined using cut-off values of <6.36 cm²/m² and <3.92 cm^2^/m^2^ for men and women, respectively [8]. Image analysis was performed using the Picture Archiving and Communication System (Konica Minolta Medical & Graphic, Inc., Tokyo, Japan), with measurements conducted in duplicate using freehand tracing; the average values were used (Figure 1). The discharge destination was classified into home and non-home categories.

Outcomes

During hospitalization, patients experienced complications other than vertebral fractures, requiring medication or consultation with other departments. Upon discharge, they were categorized into the following two groups: patients discharged home and those discharged elsewhere. Patients deemed capable of managing their daily lives at home due to alleviated lumbar back pain were discharged home, while others were transferred to facilities or supportive hospitals.

Statistical analysis

All statistical analyses were performed using the EZR platform (Saitama Medical Center, Jichi Medical University, Saitama, Japan) [9]. Qualitative data were dummy-coded as follows: sex (female, 0; male, 1), cognitive impairment (none, 0; present, 1), pre-injury mobility (ambulatory, 0; walking aid, 1; wheelchair-bound, 2), cohabitation status (no, 0; yes, 1), hospital complications (none, 0; present, 1), and sarcopenia (absent, 0; present, 1).

For the multivariate regression analysis of the length of hospital stay, explanatory variables were selected using the stepwise method with Akaike’s Information Criterion (AIC). Statistical significance was set at 5% (α=0.05), and analyses were conducted with a 95% confidence interval (95% CI).

Comparisons were performed between the discharge-to-home and non-home discharge groups, followed by a subgroup analysis of the discharge pathways. For the univariate analysis, the chi-squared test was used for qualitative data, including sex, cognitive impairment, cohabitation status, hospital complications, and sarcopenia, with α=0.05 and 95% CI. Fisher’s exact test was used to assess pre-injury mobility. Quantitative data, such as age, CCI, and CONUT score, were analyzed using the Mann-Whitney U test with α=0.05 and 95% CI. For the multivariate analysis, the stepwise method with AIC was used for variable selection, followed by logistic regression analysis with α=0.05 and 95% CI.

## Results

Patient flow

**Figure 2:**
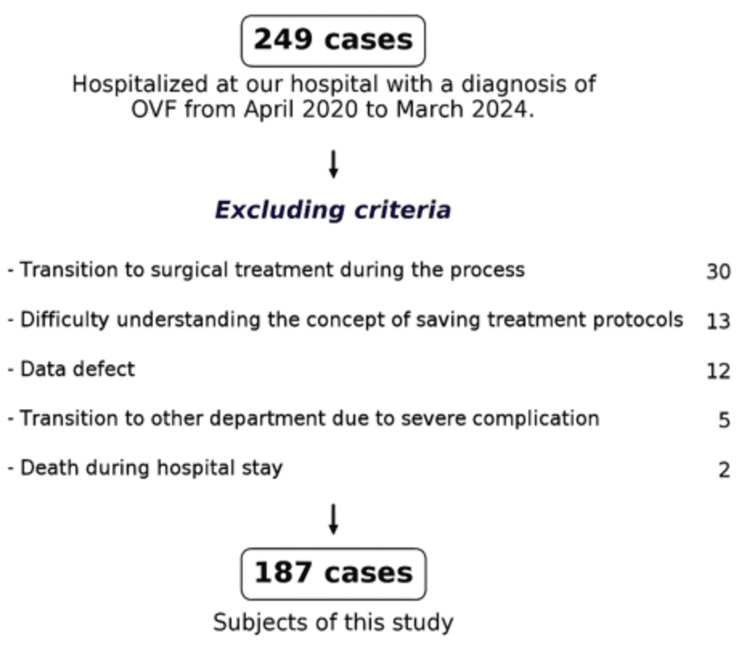
Patient flow Sixty-two patients were excluded for the following reasons: 30 patients transitioned to surgical treatment due to persistent pain and residual dynamic instability of the crushed vertebrae, 13 were discharged because of inability to understand the conservative treatment protocol owing to cognitive decline or delirium, 12 had missing data in the survey items, five required transfer to other departments and specialized intensive care due to fatal complications, and two died upon discharge due to the worsening of complications or comorbidities. OVF: osteoporotic vertebral fracture

Between April 2020 and March 2024, 249 patients diagnosed with OVFs underwent conservative inpatient treatment at our institution, of which 187 patients were included in this study (Figure 2).

No patients presented with neurological symptoms, such as lower limb weakness or bladder and rectal dysfunctions, upon admission.

Patient characteristics

Table 1 presents the patient characteristics. During hospitalization, complications occurred in 58 of the 187 patients (31.0%). Of these, urinary tract infections occurred in 25 (43.1%), and pneumonia occurred in eight (13.8%), comprising more than half of all complications (Table 2).

**Table 1:** Characteristics of patients CCI: Charlson Comorbidity Index; CONUT: Controlling Nutritional Status; SD: standard deviation

Valuable	Category	n	%	Value (mean ± SD)
Age (years)	–	–	–	81.4 ± 8.19
Sex	Female	133	71.1	–
	Male	54	28.9	–
CCI	–	–	–	6.67 ± 2.19
Cognitive impairment	Present	59	31.6	–
	None	128	68.4	–
Pre-injury mobility	Ambulatory	94	50.2	–
	Walking aid	80	42.8	–
	Wheelchair-bound	13	7.0	–
Cohabitation status	Living alone	43	23.0	–
	With others	144	77.0	–
Hospital complications	None	129	69.0	–
	Present	58	31.0	–
CONUT score	–	–	–	2.99 ± 2.23
Sarcopenia	None	60	32.1	–
	Present	127	67.9	–
Discharge destination	Home	120	64.2	–
	Non-home	67	35.8	–
Length of hospital stay (days)	–	–	–	31.0 ± 15.3

**Table 2:** Hospital complications Complications occurred in 58 (31.0%) patients during hospitalization. COVID-19: coronavirus disease

Valuable	Value
Urinary tract infection, n (%)	25 (43.1)
Pneumonia, n (%)	8 (13.8)
Electrolyte imbalance, n (%)	5 (8.6)
COVID-19, n (%)	4 (6.9)
Peripheral arthritis, n (%)	3 (5.2)
Cholelithiasis, cholecystitis, and cholangitis, n (%)	2 (3.5)
Psoas abscess, spondylitis, n (%)	2 (3.5)
Fever of unknown origin and elevated inflammatory markers, n (%)	2 (3.5)
Subarachnoid hemorrhage, n (%)	1 (1.7)
Heart failure, n (%)	1 (1.7)
Arrhythmia, n (%)	1 (1.7)
Bronchial asthma, n (%)	1 (1.7)
Dialysis shunt occlusion, n (%)	1 (1.7)
Clostridioides difficile colitis, n (%)	1 (1.7)
Malignant syndrome, n (%)	1 (1.7)

One hundred twenty patients (64.2%) were discharged home, with an average length of hospital stay of 31.0 ± 15.3 days.

Multivariate regression analysis of factors contributing to the length of hospital stay

Stepwise selection using AIC identified CCI, cognitive impairment, pre-injury mobility, cohabitation status, and comorbidities during hospitalization as explanatory variables, demonstrating overall statistical significance (F<0.001, adjusted R²=0.20).

The significant predictors of hospital stay included cognitive impairment (p=0.009), pre-injury mobility (p<0.001), cohabitation status (p<0.001), and hospital complications (p<0.001), with minimal influence from multicollinearity (Table 3).

**Table 3:** Multiple regression analysis * Significant difference. Multicollinearity among the selected explanatory variables was highly unlikely. B: partial regression coefficient; β: standardized partial regression coefficient; CCI: Charlson Comorbidity Index; SE: standard error; 95% CI: 95% confidence interval; VIF: variance inflation factor

Explanatory variables	B		β	SE	95% CI	t-value	p-value	VIF
CCI	-0.36		-0.048	0.47	–0.41 – –0.13	0.76	0.29	1.05
Cognitive impairment	5.83		0.18	2.21	1.46–10.2	2.64	0.009*	1.03
Pre-injury mobility	5.88		0.24	1.65	2.64–9.13	3.58	<0.001*	1.00
Cohabitation status	7.84		0.22	2.38	3.14–12.5	3.29	0.001*	1.04
Hospital complications	8.41		0.25	2.21	4.05–12.8	3.81	<0.001*	1.05
F-statistic	<0.001*							
Adjusted R^2^	0.20							

Subgroup analysis

In the subgroup analysis of discharge pathways, the participants were categorized into those discharged to their homes (n=120) and non-home settings (n=67). Univariate analysis revealed statistically significant differences in sex (p=0.049) and cognitive impairment (p=0.037) (Table 4).

**Table 4:** Univariate analysis *Statistically significant (p < 0.05, chi-squared test). SD: standard deviation; CONUT: Controlling Nutritional Status; CCI: Charlson Comorbidity Index

Variable	Category	Home (n=120)		Non-home (n=67)		p-value
		n	%	n	%	
Age (years, mean ± SD)	–	81.4 ± 7.98		81.1 ± 8.73		0.839
Sex	Female	79	65.8	0.049*	80.6	0.049*
	Male	41	34.2	13	19.4	
CCI (mean ± SD)	–	6.52 ± 2.08		6.88 ± 2.39		0.507
Cognitive impairment	Present	31	25.8	0.037*	41.8	0.037*
	None	89	74.2	39	58.2	
Pre-injury mobility	Ambulatory	64	53.3	30	44.7	0.530
	Walking aid	48	40.0	32	47.8	
	Wheelchair-bound	8	6.7	5	7.5	
Cohabitation status	Living alone	20	16.7	16	23.9	0.314
	With others	100	83.3	51	76.1	
Hospital complications	None	90	75.0	45	67.2	0.329
	Present	30	25.0	22	32.8	
CONUT score (mean ± SD)	–	2.90 ± 2.33		3.15 ± 2.13		0.302
Sarcopenia	None	37	30.8	23	34.3	0.743
	Present	83	69.2	44	65.7	
Length of hospital stay (days, mean ± SD)	–	26.4 ± 10.2		37.1 ± 19.0		0.165

In the multivariate logistic regression analysis using stepwise selection with AIC, sex, cognitive impairment, and cohabitation status were selected as explanatory variables. Cognitive impairment showed a statistically significant association with the discharge pathway (p=0.026). The influence of multicollinearity among explanatory variables was minimal (Table 5).

**Table 5:** Multivariate analysis (logistic regression analysis) Multicollinearity among the explanatory variables was highly unlikely; * Statistically significant. OR: odds ratio; 95% CI: 95% confidence interval; VIF: variance inflation factor

Explanatory variables	OR	95% CI	p-value	VIF
Sex	0.500	0.242–1.03	0.0615	1.17
Cognitive impairment	2.100	1.090–4.05	0.0260*	1.16
Cohabitation status	0.570	0.265–1.23	0.1520	1.06

## Discussion

To our knowledge, this is the first study to use multivariate analysis to reveal that patients’ socioeconomic background factors influence the length of hospital stay during conservative management of OVFs. Although conservative treatment usually leads to spontaneous healing with favorable outcomes, no standardized protocol has been established, and a consensus has not been reached. Some reports suggest that two weeks of bed rest significantly reduces the need for surgical intervention [4], while others indicate that avoiding bed rest in favor of early rehabilitation leads to fewer complications, lower medical costs, and shorter hospital stays [10]. Considering this, rather than enforcing a strict two-week bed rest period, we adopted a protocol where mobilization was initiated after the patient could independently turn over in bed and pain during movement subsided, or after two weeks of bed rest, whichever occurred first. Meanwhile, surgery is required in 15-35% of cases due to vertebral instability, persistent back pain, or neurological symptoms [2,3]. Prognostic factors for poor outcomes include localized high-signal or diffuse low-signal intensity on MRI T2-weighted images, which indicate a high risk of developing pseudoarthrosis [11].

Research on factors such as cognitive function and living environment remains inconclusive, and studies on the duration of hospitalization and discharge destination for OVFs are sparse. A study reported that patients with lower grip strength had significantly longer hospital stays [12]. Sarcopenia significantly affects the Barthel index at the first visit and discharge, with patients with sarcopenia showing a significantly higher rate of discharge to nursing homes [13].

Our study investigated factors contributing to complications during hospitalization, including pre-injury mobility, cohabitation status, cognitive impairment, and length of hospital stay, which have not been previously explored together. Although discussions on intensive care methods have been extensive, they lack systematic establishment [4,14-16]. Reports have indicated a significant increase in complications, such as urinary tract infections [17] and respiratory complications [18] during hospitalization for OVFs. In our study, more than half of the complications were attributed to urinary tract infections and pneumonia or upper respiratory tract infections. Factors such as prolonged urinary catheterization, disuse-related decline in swallowing function, and reduced oral intake during bed rest may contribute to these complications. Regarding discharge pathways, a significant increase was found in facility discharge among patients with sarcopenia [13]. No previous research has explored the relationship between cognitive function and discharge pathways. However, patients with dementia experience significant cognitive decline during hospitalization, which warrants attention [19].

When complications arise during hospitalization, concurrent therapy for these complications alongside OVF treatment and rehabilitation becomes necessary. In particular, consumptive complications, such as infections, pose a risk to the patient’s overall physical condition and stamina, potentially impeding OVF treatment and rehabilitation. Therefore, referring to other specialties or specialized therapeutic interventions can further prolong this process.

Several factors, such as pre-injury mobility, living arrangements, and cognitive function, significantly influence discharge decisions. When issues arise that cannot be solely addressed by orthopedic care, the duration of hospital stay may lengthen, necessitating arrangements for discharge to locations other than the patient’s home. Multidisciplinary collaboration is also crucial for shortening the length of hospital stays. Reports indicate that the involvement of multiple disciplines and liaison services helps reduce hospitalization in patients with osteoporotic proximal femoral fractures [20]. Similarly, interventions by geriatricians and liaison services contribute to shorter hospital stays and reduced healthcare costs among patients with non-vertebral fractures of ≥65 years of age [21].

Preventive measures and management of in-hospital complications, such as continence management by urinary care teams, dietary modifications by nutritionists, and enhanced swallowing-function assessments and training by speech therapists, may also contribute to reducing hospitalization duration. In cases involving cognitive impairment, arranging discharge to locations other than home may require additional time. Early and robust discharge support under the supervision of social workers could reduce the length of hospital stay. Furthermore, conducting objective cognitive assessments for all patients upon admission and continuing regular post-admission evaluations could assist in shortening the length of hospital stay.

We successfully identified several relevant factors contributing to the length of hospital stay in patients undergoing treatment for OVFs. Implementing early interventions involving a multidisciplinary approach based on these factors is essential to increase the likelihood of discharge to home. This approach can shorten the length of hospital stays, reduce medical costs, and extend healthy life expectancy.

This study has some limitations. Clinical symptoms, such as pain, which are expected to impact the length of hospital stay significantly, and imaging findings related to fracture severity were not evaluated due to the retrospective observational nature of this single-center study. Since our conservative treatment protocol initiated mobilization only after the resolution of pain during bed rest, these factors may have influenced the timing of mobilization and length of hospital stay; however, their impact remains unclear. Additionally, the adjusted coefficient of determination (R²) was low (0.20), suggesting considerable data differences in some variables and the potential presence of unaccounted factors influencing the length of hospital stay. The influence of social factors, which are difficult to quantify, also represents a limitation of this study. Furthermore, the sample size may be insufficient for robust multivariate analysis. Future studies with a larger sample size, along with prospective validation, are warranted.

Among the in-hospital complications, four cases of coronavirus disease infection required a seven-day isolation period. Since all four cases tested positive upon admission, isolation was implemented during the initial bed rest period. Although the impact on rehabilitation progress was considered minimal, the potential effects on hospital stay due to disuse syndrome or muscle weakness cannot be ruled out and remain unclear.

Many patients who were evaluated as having cognitive decline during admission showed further deterioration in cognitive function during hospitalization. However, this could be managed through medication adjustments, and it did not significantly affect rehabilitation; therefore, these cases were not included as hospital complications. Cognitive decline during hospitalization was also considered a distinct condition from narrow-sense dementia, as it could have been delirium, a transient disturbance of consciousness associated with hospitalization. Since distinguishing this from the exacerbation of pre-existing dementia proved challenging, these cases were not included as hospital complications in this study.

Regarding analgesics, the type, dosage, and duration of administration varied depending on the patient’s pain level and the attending physician’s discretion. Non-steroidal anti-inflammatory drugs were prescribed to patients without renal dysfunction and acetaminophen to those with renal dysfunction, with the appropriate dosage adjusted based on body weight. However, since this study is a retrospective analysis and the prescription of analgesics was not standardized, the impact on the length of hospital stay remains unclear.

## Conclusions

Inpatients with OVFs undergoing conservative treatment had a significantly longer hospital stay if they had cognitive impairment, lower pre-injury mobility, lived alone, or experienced complications during hospitalization. Patients with cognitive impairment were significantly less likely to be discharged home but more likely to be discharged to non-home settings, such as care facilities. Hospital-acquired complications such as urinary tract infections and pneumonia accounted for more than half of all cases. Cognitive function influenced discharge outcomes. Furthermore, collaborative interventions by a multidisciplinary team targeting these factors could increase home-discharge rates, consequently reducing the length of hospital stay, reducing medical costs, and extending healthy life expectancy. In the future, we intend to design prospective studies focusing on the contributing factors identified in this study to shorten the length of hospital stay.
